# A unique pre-endothelial layer at the posterior peripheral cornea: ultrastructural study

**DOI:** 10.1038/s41598-022-06552-6

**Published:** 2022-02-15

**Authors:** Saeed Akhtar, Ramachandran Samivel, Adrian Smedowski, Aljoharah Alkanaan, Ali Masmali, Omar Kirat, Adnan Ali Khan, Turki Almubrad

**Affiliations:** 1 Cornea Research Chair, Department of Optics and Vision Science, College of Applied Medical Sciences, King Saud University, P.O. Box 10219, Riyadh, 11433 Saudi Arabia; 2 College of Applied Medical Sciences, Inaya Medical College, Riyadh, Saudi Arabia; 3 Department of Physiology, Faculty of Medical Sciences in Katowice, Medical University of Silesia, Katowice, Poland; 4 Department of Ophthalmology, King Khalid Eye Specialist Hospital, Riyadh, Saudi Arabia

**Keywords:** Anatomy, Medical research

## Abstract

This study was conducted to investigate the ultrastructure of a unique structures at the anterior side of the endothelium of the posterior peripheral cornea and compare their inner fibers to those of the limbus and sclera. The unique structures at the anterior side of endothelium was referred as a pre-endothelial (PENL) structures in the present manuscript. Ten anonymous-donor human corneoscleral rims (leftover after corneal transplants) were processed for electron microscopy. Semi-thin sections were examined using an Olympus BX53 microscope, and ultrathin sections were studied using a JOEL 1400 transmission electron microscope. A unique PENL structures was identified at the posterior peripheral cornea at a radial distance of approximately 70–638 µm, from the endpoint of Descemet’s membrane. The PENL thinned out gradually and disappeared in the center. The contained an electron-dense sheath with periodic structures (narrow-spacing fibers), wide-spacing fibers, and numerous microfibrils. Typical elastic fibers were present in the sclera and limbus but were not observed in the PENL. This study revealed the existence of a new acellular PENL, containing unique fibrillar structures that were unseen in the corneal stroma. From the evidence describe in this paper we therefore suggest that PENL is a distinct morphological structure present at the corneal periphery.

## Introduction

The eyeball’s outermost fibrous tunic is the cornea and sclera, which are soft connective tissues designed to provide structural integrity of the globe and protect the eye’s inner components from pathological insults^1^. The clear transparent cornea extends to the Descemet’s membrane endpoint (DME) in the corneolimbal junction. Detailed ultrastructural studies have shown that the human cornea is composed of five layers: epithelium, Bowman’s layer, stroma, DM, and endothelium (EN)^2–5^. A substantial proportion of the cornea consists of the stroma, which constitutes 90% of human corneal thickness. The stroma’s main components are collagen fibrils organized uniformly in parallel-running lamellae to provide transparency to the cornea. The unique spatial arrangement of the collagen fibers (CFs) is governed by interfibrillar macromolecular glycol conjugates, known as proteoglycans^6–8^.

Dua et al.^9^ have reported the existence of a sixth layer in the pre-DM region in the human cornea based on observations from a “big bubble” corneal transplant technique^9^. This layer was composed of 5–8 lamellae of collagen fibrils with a diameter of 21.70 ± 2.43 nm and spacing of 9.64 ± 7.74 nm, running transverse, longitudinal, and oblique^10^. The existence of a distinct pre-DM layer has not been reported previously in studies on the architecture of the cornea^5^. Schrehardt et al.^5^ have discussed the behavior of the stroma using the “big bubble” technique and reported significant variations in the distance of keratocytes from DM at the center, mid-periphery, and peripheral stroma^5^. At the DM–stroma interface, randomly running collagen fibers form a thin layer instead of the presence of acellular pre-DM layer (PDL) as described by Dua et al.^9^ Chen et al.^11^ have discovered the presence of hyper reflective lattice structures at the interface between the EN and DM and dome-shaped basolateral endothelial cells.

The ultrastructure of the posterior stroma in normal and pathological human corneas has been described previously^12–14^. Feneck et al.^14^ have shown the banded and elastic fibers above DM in the peripheral cornea and identified them using tannic acid with glutaraldehyde^14^. The presence of microfibrils, long-spacing collagen in the basement membrane in the posterior collagenous layer, has been shown in bullous keratopathy, Fuchs’ dystrophy, and superficial hypertrophic dendriform epitheliopathy^12,15,16^. Strong labeling of the TGFBI/BIGH3 was observed in DM and microfibrils but not on the long-spacing collagen fibrils of the posterior collagenous layer (PCL)^15,17^.

No published study has described the fibrous structure of the DM–EN interface of the posterior peripheral cornea in a normal human cornea. In this study, we investigated the structure of the DM–EN interface of a posterior peripheral normal human cornea from the DME. The ultrastructure of the fibers at the radial distance from the DME of the posterior peripheral cornea is described here in detail, and the similarities and differences of the sclera, limbus, and trabecular meshwork fibers were examined.

## Results

### Pre-endothelial structures at the posterior peripheral cornea

**Figure 1:**
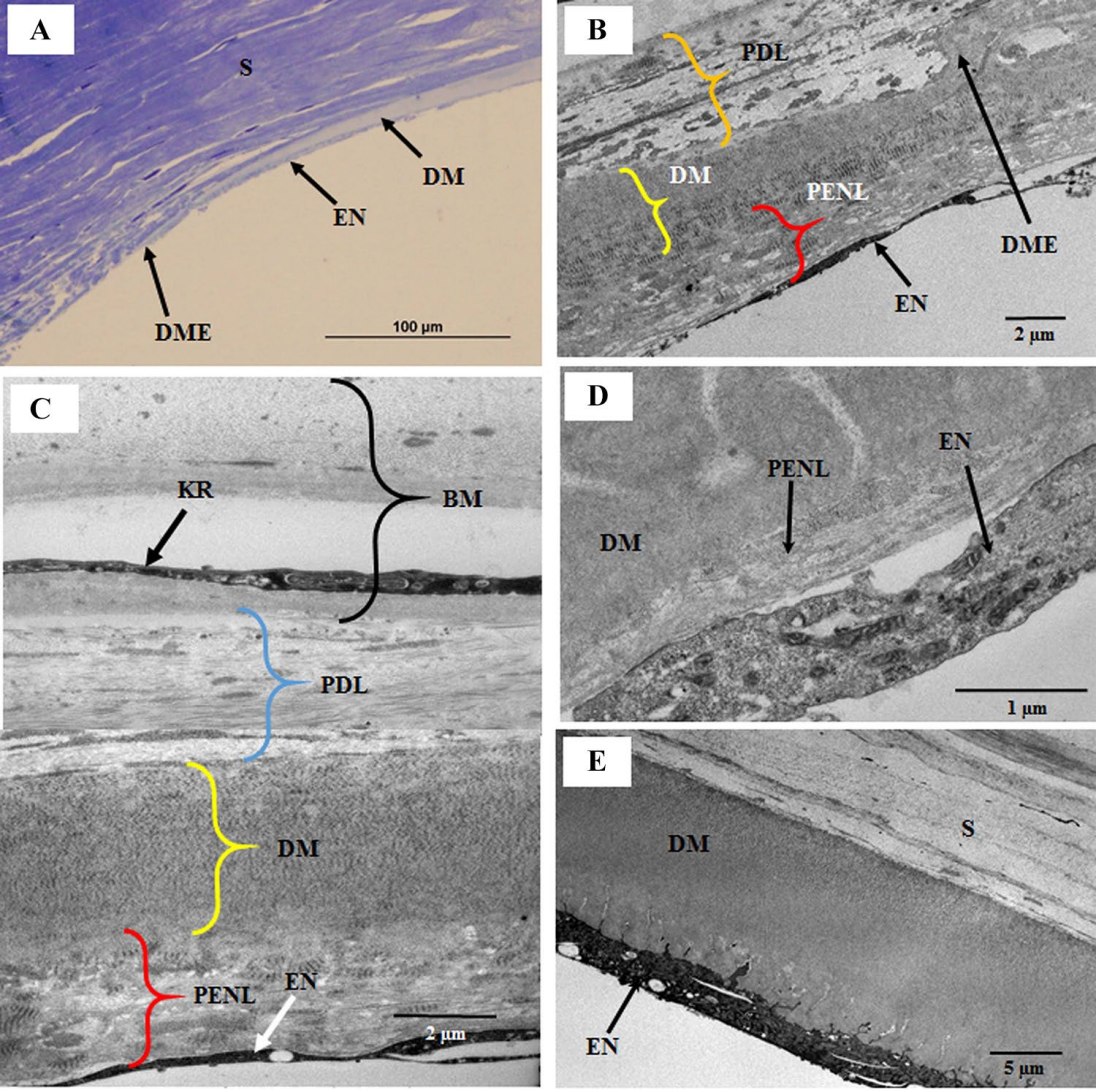
Light and electron micrographs of the peripheral posterior cornea; (**A**) Light micrograph of a part of the peripheral cornea showing the stroma, Descemet’s membrane (DM), endothelium (EN), and the Descemet’s membrane endpoint (DME). (**B**) Part of the peripheral cornea at a distance 105 µm from the DME, showing the DME, EN, pre-endothelial layer (PENL), DM, and pre-DM layer (PDL); (**C**) Part of the peripheral cornea at a distance of 167 µm from the DME, showing the EN, PENL, DM, PDL, and split basement membrane containing a large cell. (**D**) Part of the posterior cornea at the distance of 452 µm from the DME, showing the thinning of the PENL between DM and the EN. (**E**) Part of the posterior cornea at 761.6 nm from the DME, showing the stroma, DM, and EN. No PENL and PDL are observed. *BM* basement membrane, *DM* Descemet’s membrane, *DME* Descemet’s membrane endpoint, *EN* endothelium, *KR* keratocyte, *PDL* pre-Descemet’s membrane layer, *PENL* pre-endothelial layer, *S* stroma.

A unique PENL structure on the anterior side of the endothelium was present at the posterior peripheral cornea at a radial distance of 70–638 µm from the DME (Fig. 1A–D). Three layers—PDL, DM, EN and acellular PENL structures between DM and the EN,—were identified at the posterior peripheral cornea (Table 1; Fig. 1B–E). We viewed the cornea as we approached it from the center; the posterior peripheral cornea contained four layers—a split layer of homogenous material (basement membrane) containing a large keratocyte with a large nucleus, PDL, DM, EN and PENL structures (Table 1; Fig. 1C). Furthermore, the split basement membrane united and appeared as a single basement membrane. Then, they moved toward the central cornea, the PDL disappeared, and the PENL structures thinned down, leaving only two layers—DM, EN, and PENL structures (Table 1; Fig. 1D). After a distance of 638 µm from the DME, the PENL structures also disappeared, leaving only DM and the EN (Table 1; Fig. 1E).

**Table 1:** The number and name of the Layers present at a distance from the DME at the posterior pre-limbal cornea.

Distance from DME (µm**)**	Number of layers	Name of the layers
70		PENL emerge
105	4 layers	PDL, DM, PENL, EN
167	5 layers	Split BM, PDL, DM, PENL, EN
245	5 layers	United BM, BM, PDL, DM, PENL, EN
511	5 layer	DM, Thin PENL, EN
638	2 layers	DM, EN

### Ultrastructure of PENL fibers at the posterior peripheral cornea

**Figure 2:**
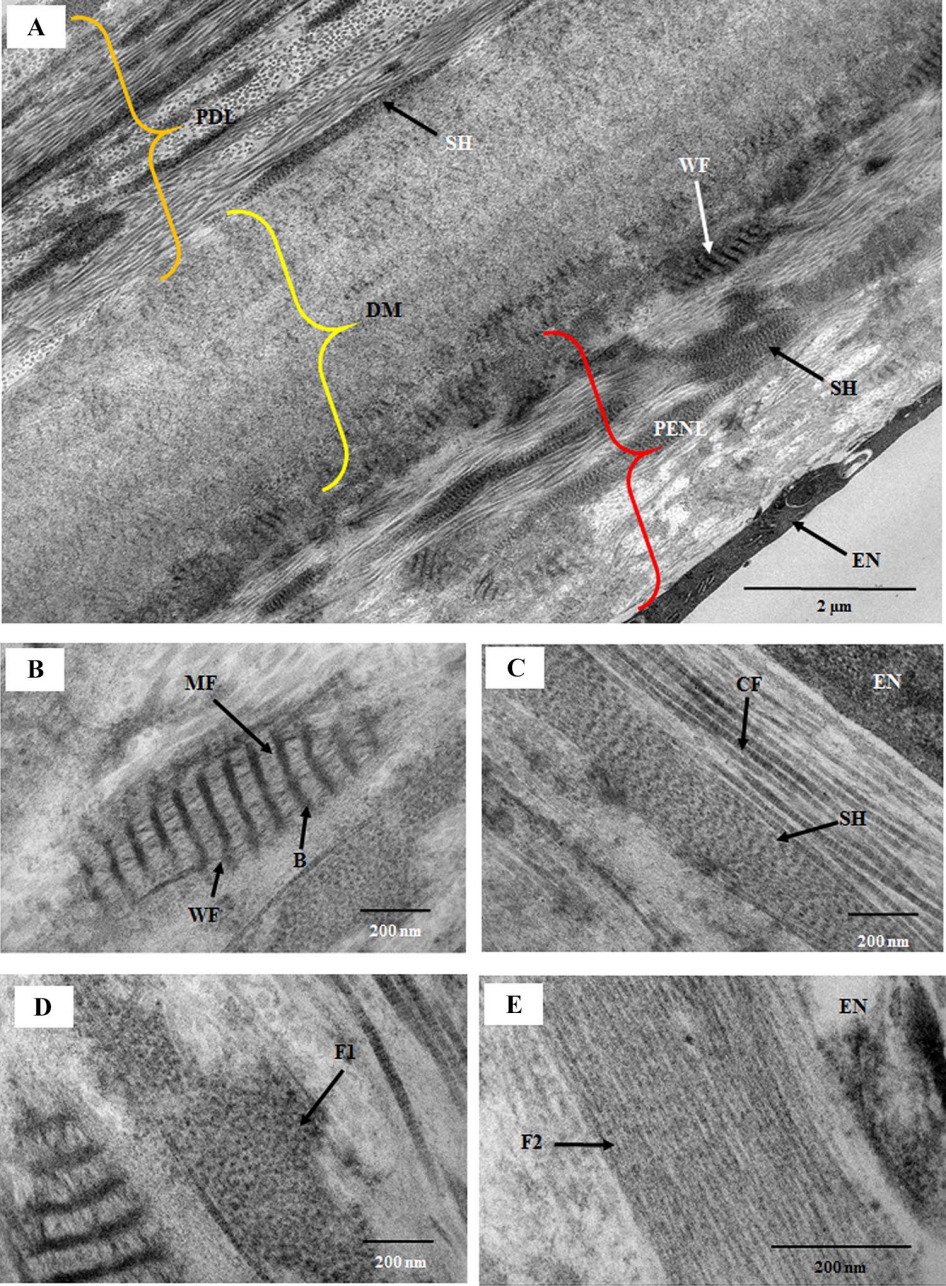
Electron micrograph of the posterior peripheral cornea. (**A**) Part of the posterior peripheral cornea, showing the endothelium (EN), pre-endothelial layer (PENL), Descemet’s membrane (DM), and pre-Descemet’s membrane layer (PDL). The PENL and PDL contained wide-spacing collagen, aggregates of microfibrils, and sheath material, consisting of electron-dense transverse bands (narrow-spacing fibers). DM had aggregates of the wide-spacing fibers located at its posterior surface. The major part of DM had a banding pattern. (**B**) Part of the PENL showing wide-spacing fibers containing dark bands (37.52 ± 4.99 nm) with a periodicity of 67.89 ± 5.91 nm, and the bands were connected by thin microfibrils (15.19 ± 3.00 nm). (**C**) Part of the PENL showing a sheath material consisting of electron-dense transverse bands forming a lattice. The sheath might be the outer part of the “elastic-like fibers” described by Lutjen-Drecoll et al.^18^ (**D**) Part of the PENL showing a cross-section of microfibrils (F1) with a diameter of 16.13 ± 4.24 nm. (**E**) Part of the PENL showing longitudinally running thin fibrils (F2) with a thickness of 10.14 ± 2.70 nm, just above the EN. These fibrils were described as fibrils of oxytalan fibers by Carrington et al.^19^. *B* electron-dense bands; *CF* collagen fibril, *DM* Descemet’s membrane, *EN* endothelium, *F1* fibrils with a thickness of 16.13 ± 4.24 nm, *F2* fibrils with a thickness of 10.14 ± 2.70 nm, *MF* microfibrils, *NB* narrow bands, *PDL* pre-Descemet’s membrane layer, *PENL* pre-endothelial layer, *SH* sheath material consisting of electron-dense bands (narrow banded fiber), *WF* wide-spacing fibers.

**Figure 3:**
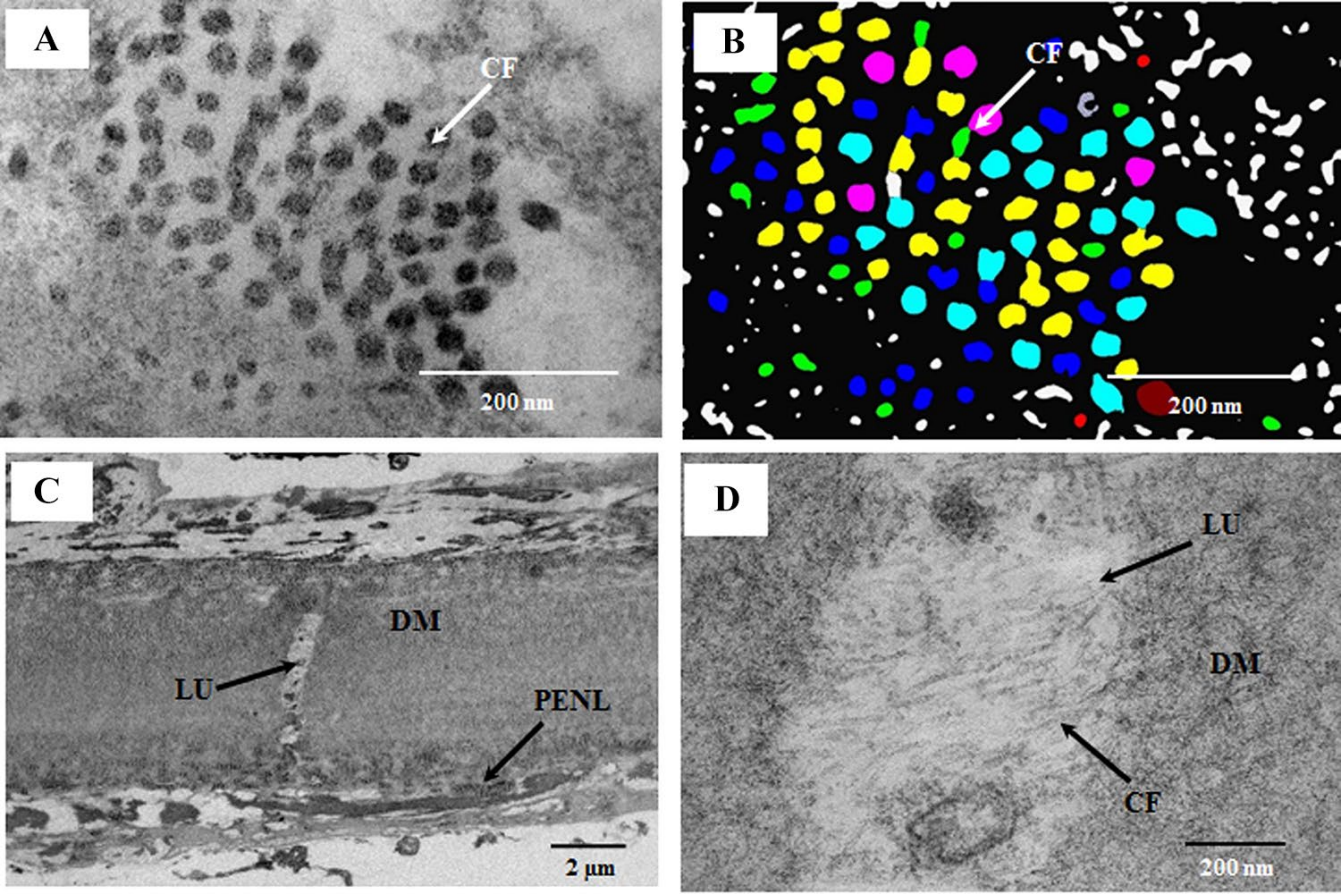
Electron micrographs illustrate the fibers of the pre-endothelial layer (PENL), pre-Descemet’s membrane layer (PDL), and Descemet’s membrane (DM). (**A**) Part of the PENL containing collagen fibrils (CFs) near the endothelium (EN). (**B**) Digital color-coded image of CFs showing fibrils ranging from 15 to 45 nm in diameter. (**C**,**D**) Part of DM containing electron-lucent space that includes CFs. *CF* collagen fibril, *DM* Descemet’s membrane, *LU* lucent space, *PENL* pre-endothelial layer. Red, 15–20 nm; green, 20–25 nm; blue, 25–30 nm; yellow, 30–35 nm; terracotta, 35–40 nm; orange, 40–45 nm.

The thickness of the PENL band ranged from 1.01 ± 0.1 to 3.01 ± 0.26 µm, and the PENL band was at its thickest at a distance of 113 µm from the DME. The PENL contained CFs, long fibers, wide-spacing fibers, and a sheath with periodic bands (17.82 ± 2.59 nm) (Fig. 2A–E). The wide-spacing fibers contained electron-dense bands with a mean diameter of 37.52 ± 4.49 nm, which enclosed the electron-lucent space (67.89 ± 5.91 nm), and were connected with each other by thin microfibrils (15.19 ± 3.0 nm) (Fig. 2B). Numerous banded collagen fibrils were present around the EN, and a sheath with periodic structures looked similar to the structure of the narrow-spacing fibers with thin bands (17.82 ± 2.59 nm) (Fig. 2C). Numerous aggregates of cross-sections of microfilaments (17.82 ± 2.59 nm) and longitudinally running fibrils (10.71 ± 1.64 nm) were observed in the PENL near the EN (Fig. 2D,E). The CFs near the EN were in non-uniformly distributed with a mean diameter of 28.99 ± 6.7 nm and inter-fibrillar spacing of 47.41 ± 15.5 nm (Fig. 3A,B). The color coding of the collagen fibrils according to their diameter showed that the diameter ranged from 20 to 45 nm (red color to orange color). DM above the PENL contained wide-spacing fibers at both its anterior and posterior faces and electron-lucent spaces within DM containing thin fibrils (Fig. 3C,D).

### Ultrastructure of elastic fibers, banded fibers, and microfibrils of the sclera and limbus

**Figure 4:**
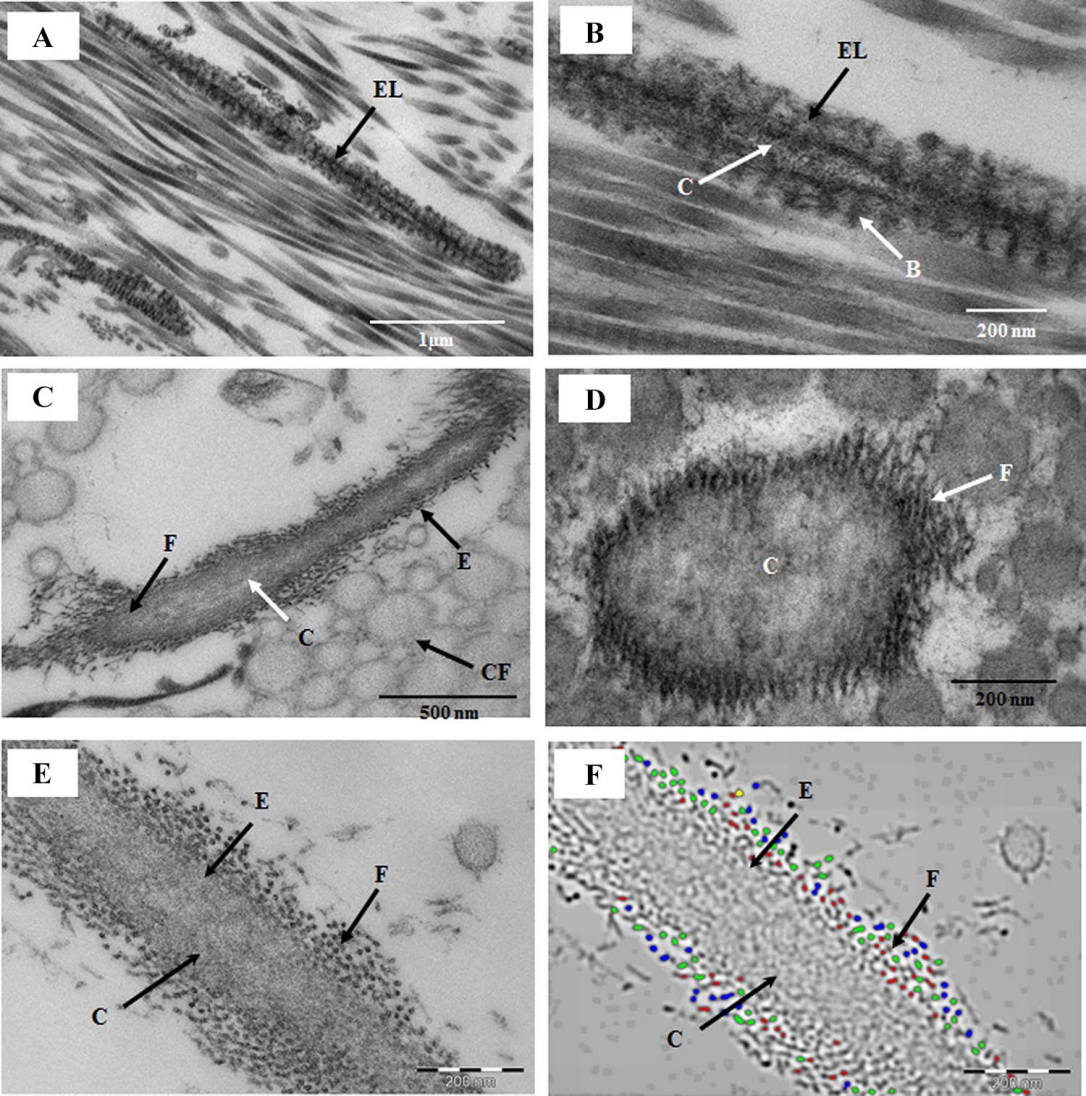
Electron micrographs of the fibers of the sclera. (**A**,**B**) Part of the sclera showing a longitudinally running elastic-like fiber (EL) containing a central elastic core (C) and thick electron-dense bands (B), which are connected by thin microfibrils. These fibers are similar to EL fibers reported by Rohen et al.^20^ (**C**) Longitudinally running typical elastic fibers containing a homogenous central core, surrounded by thin microfibrils (6.78 ± 2.70 nm). (**D**) Cross-section of the elastic fibers showing a central core surrounded by thin fibrils. (**E**) Part of elastic fibers containing an elastic core and cross-section of microfibrils. (**F**) Digital image of thin fibrils (6.79 ± 2.70 nm) around the elastic core of the elastic fiber. *EL* elastic-like fibers (Rohen et al., 1981), *E* elastic fiber, *F* fibrils (6.79 ± 2.70 nm), *C* elastic core, *B* electron-dense bands; *CF* collagen fibril.

The ultrastructure of the elastic fibers in the sclera and limbus were similar to each other. Depending on the plane of sectioning, the structure of the elastic fibers varied at different parts of the sclera and limbus. Mainly, two types of fibers were present in the sclera and limbus (Fig. 4A–F). The first type of fiber was long banded fibers (242.62 ± 23.31 nm,) containing electron-dense spaces (35.95 ± 3.85 nm) with periodicity of approximately 47.30 ± 3.85 nm (Fig. 4A,B). The electron-dense bands were connected to each other by microfibrils (9.17 ± 1.04 nm), and the central part of the fibers contained a tubular electron-lucent central core with a diameter of 53.336 ± 7.71 nm, which was interrupted by electron-dense strands (Fig. 4B). The second type of fiber was typical elastic fibers composed of a homogenous central elastic core surrounded by thin fibrils (Fig. 4C,D). Digital analysis of the fibrils showed that the diameter of these fibrils was approximately 6.79 ± 2.70 nm (Fig. 4E,F) and that the fibrils were fibrillin fibrils based on their thickness. The homogenous central core of the elastic fibers contained electron-dense particles (Fig. 4C,D).

## Discussion

**Figure 5:**
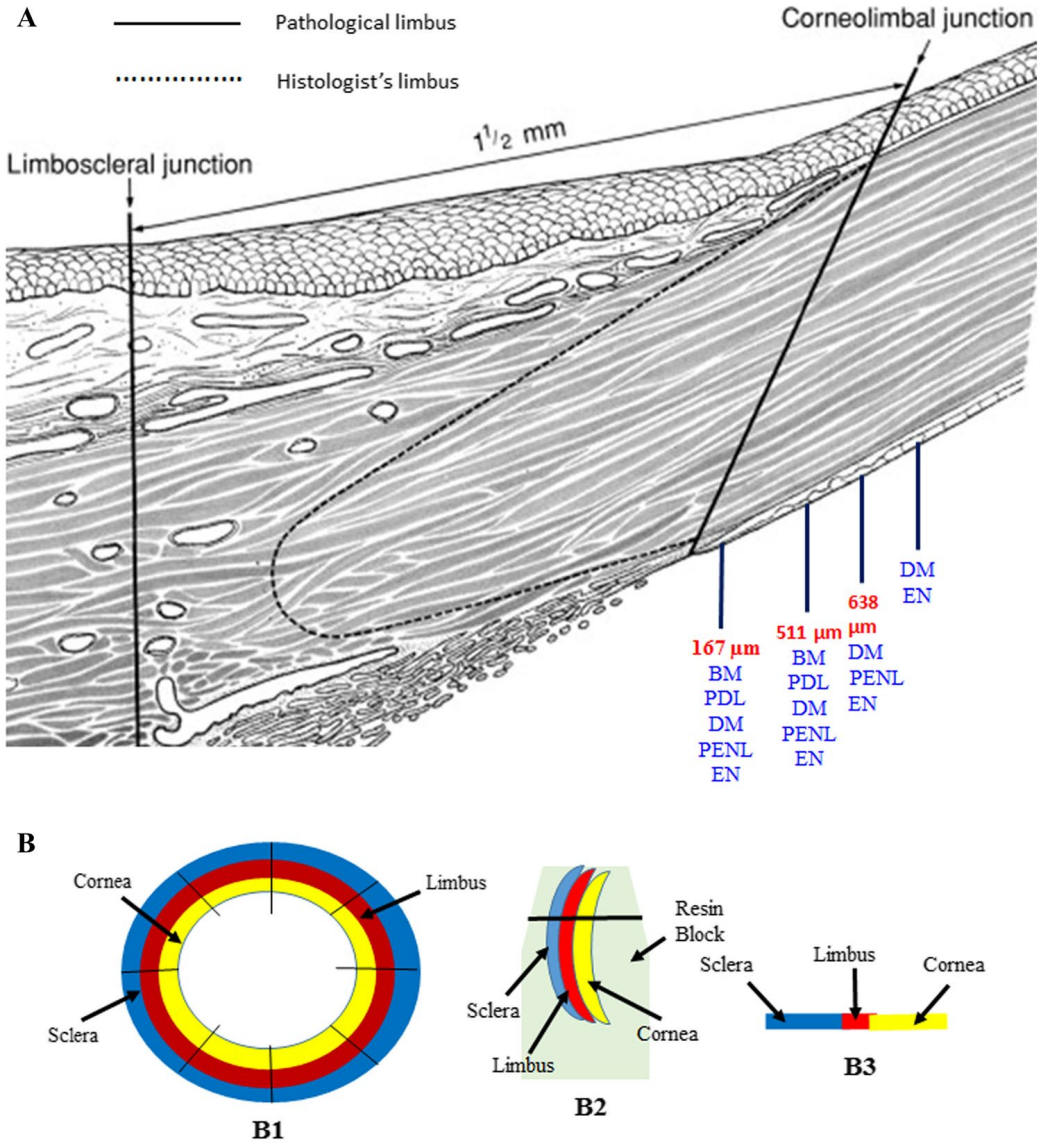
(**A**) A Schematic illustration of the posterior peripheral cornea showing the distribution of the layers according to the distance from the DME. (**B**) A schematic diagram of the corneoscleral rims (Modified from Hogan et al.)^21^. (**B1**) The corneoscleral rims cut into small pieces to embed in the resin to make blocks. (**B2**) Block containing the sclera, limbus, and cornea for sectioning. (**B3**) A semi-thin section from the block containing the sclera, limbus, and cornea. *BM* basement membrane, *BW* Bowman’s layer, *DM* Descemet’s membrane, *DME* Descemet’s membrane endpoint, *EN* endothelium, *PDL* pre-Descemet’s membrane layer, *PENL* pre-endothelial layer, *S* stroma, *TB* trabecular meshwork.

Changes in the structure of the posterior part of the cornea are observed from the center to the peripheral cornea, especially near the corneolimbal junction (CLJ). At the CLJ, DM originates as a curve and is surrounded by connective tissue at both the anterior and posterior sides (Fig. 1B,C). This study revealed that PENL structures exists between DM and the EN at the peripheral cornea. The PENL emerges at a distance of 70 µm from the DME and runs up to 638 µm at the peripheral cornea. Initially, the posterior peripheral cornea contained a split basement membrane, PDL, DM, PENL, and EN. Gradually, the basement membrane and PDL disappeared, leaving the PENL, DM, and EN. Finally, the PENL also disappeared, leaving DM and the EN, at a distance of 638 µm away from the DME (Fig. 5A). There was no variation in structure of PENL with respect to the ages of the samples.

Dua et al.^10^ have reported that wide-spacing or long-spacing fibers observed near the banded DM were similar to the wide-spacing fibers in the trabecular meshwork^10^. Feneck et al.^14^ have assessed the distribution of elastic fibers in mouse and human peripheral cornea and have shown the presence of elastic fibers at the PDL or anterior to DM, whereas Dua et al.^10^ did not find any elastic fibers in the PDL^10^. Lewis et al.^13^ have reported the arrangement of the banded fibers in the human central and peripheral cornea and suggested that these banded fibers are elastic fibers^13^.

Typical elastic fibers containing a central elastic core surrounded by the microfibrils observed in the sclera and limbus were not observed in the PENL and PDL in this study. These normal elastic fibers have been described in the normal human eye, heart valve, and intervertebral disc^20,22,24,25^. We observed these elastic fibers in the sclera, but not in the PDL and PENL. The PDL contained many fibers running parallel to DM, which were surrounded by collagen fibrils. These fibers contained a homogenous granular material or lucent area, whereas others had banding patterns. The long thick fibers running parallel to DM contained a banded collagen fibril in the central cavity of the fiber. It could be possible that the long thick fibers are an aggregation or fusion of the banded collagen fibrils.

The PENL contained wide-spacing fibers, narrow-spacing fibers, aggregates of microfilaments, and collagen fibrils observed near the EN, with a diameter of 28.99 ± 6.8 nm. This was similar to the stromal CF diameter reported previously^26^. Several fibers composed of tubular microfilaments distributed were observed near the EN. These fibers with tubular microfilaments have been observed in cat cornea and are considered oxytalan fibers^19,27^. Lutjen et al.^18^ have described wide-spacing fibers as *Gitterkollagen* (curly or lattice collagen) in a human trabecular meshwork^18^. The fibers consisted of electron-dense longitudinal and transverse portions with a periodicity of 100 nm. It was suggested that the fine fibrillar material was made of collagen, whereas the electron-dense material around the fine fibrils was chondroitin sulfate. The component of the transverse band could not be identified by the authors. In this study, the wide-spacing fibers observed in the PENL had a structure similar to that of *Gitterkollagen* fibers found in the trabecular meshwork described by Lutjen et al. ^18^ Rohen et al.^20^ have described “elastic-like fibers” in corneoscleral lamellae, which consisted of an electron-dense central core with light strands, surrounded by a sheath with periodic structures^20^. It was suggested that the “elastic-like fibers” are different from typical elastic fibers^18,20^. The narrow-spacing fibers observed in this study had a structure similar to that of the sheath of the “elastic-like fibers” of the trabecular meshwork reported by Lutjen et al.^18^.

The wide-spacing fibers were similar to the fibers in the PENL and trabecular mesh work and were not present in the PDL. These fibers were present at the surface of the anterior banded layer of DM (ABL) and posterior non-banded layer of DM (PNBL). The middle part of DM contained lucent spaces containing small fibrils with a banding pattern. These fibrils might be at an early stage of CFs, which migrate from DM to the PDL and PENL. It is possible that wide-spacing fibers, CFs, and other fibers are produced in DM. Dua et al.^10^ have reported that fibers are produced in the PDL and migrate from the PDL to the trabecular meshwork, whereas Fenwick et al.^14^ have suggested that fibers migrate from the trabecular meshwork to the PDL. In our observation, no similarities between PDL and trabecular meshwork fibers were observed; instead, similarities between the fibers of the PENL and trabecular meshwork were found. Considering the similarities between the fibers of the trabecular meshwork (TM) and PENL, we believe that the PENL and TM may have been from the same source of origin. We suggest the source of origin could be morphological residue from embryonic development of corneal endothelial (CE) and TM structures.

Fibers similar to the fibers of the PENL, were observed in the Fuchs dystrophy and bullous keratopathy. Fuchs dystrophy is progressive degeneration of the corneal stroma, thickening of the Descemet’s membrane and degeneration of endothelium. The posterior part of the Descemet’s membrane contained collagenous layer^11^. Yuen et al.^11^ have described it as a posterior collagenous layer (PCL) as a part of DM in Fuchs’ dystrophy, whereas Akhtar et al.^14^ have described the PCL as a separate layer from DM in bullous keratopathy cornea.

The PENL is a part of the posterior peripheral cornea and is composed of unique fibers and fibrils, which are not present in the central corneal stroma. The ultrastructural knowledge of PENL will help us understand the pathology of Fuchs dystrophy and bullous keratopathy. Further studies are required to investigate the biochemical composition of the PENL.

The ultrastructure of the epithelium, stromal collagen fibrils, proteoglycans, Descemet’s membrane and endothelium the samples were intact and were similar to those in the fresh donor cornea describe in the previous studies. We believe that the storage of the corneoscleral rims in the in standard (Optisol-GS) organ culture medium did not affect the ultrastructure of the peripheral cornea. It would be interesting to study the structure of PENL in fresh donor cornea to compare its structure with that the organ culture cornea.

There is growing evidence that a transition zone or Schwalbe’s Ring region between the EN and TM contains adult stem cells/progenitor cells called Schwalbe's line cells^28–30^. Yu et al.^31^ called these cells “PET cells” (Progenitors for Endothelium and Trabeculum) and these cells have the ability to supply new cells to the CE and TM and replace their non-functional or lost cells^31^. Our finding of a PENL could be considered as morphological residue from the embryonic development of corneal endothelial (CE) and trabecular meshwork (TM) structures which are produced from mesenchymal embryonic cells. We believe that the PENL may have developed, creating an interface between EN and TM and forming a transition zone. The PENL may also contain stem cells or progenitor cells similar to the transition zone or Schwalbe’s Ring region which could have a role in replacing non-functional cells of EN. Further research is required to explore this mechanism.

## Materials and methods

### Collection of human corneoscleral rims

Ten healthy anonymous-donor human corneoscleral rims (leftovers from corneal transplants) were obtained from eye bank. The corneo-scleral rim were stored in standard (Optisol-GS) organ culture medium. We do not have any information about duration of the storage of the cornea. The donors’ ages ranged from 21 to 75 years. Ethical approval for corneal tissue procurement and use was granted by the Local Ethical Committee of King Saud University, Saudi Arabia. All experiments were conducted according to the guidelines of “Standing Committee for Research Ethics on Living Creatures (SCRELC),” Saudi Arabia (policy available at: https://www.uod.edu.sa/sites/default/files/resources/implementing_regulations_0.pdf).

### Sample processing

The peripheral part of the donor corneoscleral rims was left over after the transplantation of the central cornea, fixed immediately in 2.5% glutaraldehyde in 0.1 M phosphate buffer at pH 7.0 for 2 h. The corneoscleral rims, which contained the cornea, limbus, and sclera (Fig. 5B1), was cut into 1.5 mm pieces, and five pieces from each ring were fixed again in 2.5% glutaraldehyde in 0.1 M phosphate buffer at pH 7.0 for 2 h and then washed three times with 0.1 M phosphate-buffered saline (15 min × 3). The pieces from each corneoscleral rims were processed separately in individual vials. The pieces were dehydrated in a graded series of ethanol (70%, 90%, and 100% twice) for 15 min each, followed by dehydration in acetone (100% twice) for 30 min each. The tissue was infiltrated in a mixture of acetone + resin overnight, followed by 100% resin 8 h × 3. The pieces were embedded in Spurr’s resin (100%). During embedding, the corneoscleral rims pieces were placed into an embedding mold with the sclera, limbus, and peripheral cornea facing the sectioning surface (Fig. 5B2). The tissue was polymerized in Spurr’s resin at 70 °C for 8 h in the form of blocks. Fifty blocks were prepared for sectioning.

### Ultra-cut microtome sectioning

The blocks were sectioned using RMC ultra-cut microtome (Reichert-Jung Ultra-cut Microtome) to acquire semi-thin (0.5 µm) cross-sections. The semi-thin cross-sections were approximately 1 mm wide, containing the limbus, corneolimbal junction (CLJ), and peripheral cornea (Fig. 5B3). The semi-thin sections were collected on glass slides and stained with toluidine blue for 30 s and washed with water. The slides were observed under a BX53 light microscope, and the DME was located.

Once the DME was located on the light microscopic sections (Fig. 5B3), the block was further trimmed from the sides to make the cutting surface approximately 0.5 mm wide to obtain ultrathin Sects. (75 nm). Fifteen ultrathin sections were cut from each block, making 750 sections from 50 blocks. The ultrathin sections were collected on 100 mesh, 200 mesh, and slot grids coated with formvar. The 100 mesh and slot grids were used to keep the CLJ from falling across the grid bars. A major challenge faced in collecting the sections was preventing the DME from falling over the grid bar of the 100 and 200 mesh grids. It was challenging to get the DME, DM, PENL structure, and EN on the space between the grids to observe the structures.

### Transmission electron microscopy imaging and software analysis

Ultrathin sections were collected on copper grids and stained with 2% uranyl acetate (10 min) and lead citrate (10 min) separately. They were washed and observed using a transmission electron microscope JEOL 1400 (JEOL, Akishima, Japan). Either some sections were broken under the electron beam or the PENL was shadowed by the grid bar. The blocks were cut again to get suitable sections that would better help identify the PENL, which was difficult to detect. Digital images of the CLJ and peripheral posterior cornea were captured using a bottom-mounted 14-megapixel Quamesa camera and iTEM Soft Imaging System (Soft Imaging System, Munster, Germany). The microfilaments, wide-spacing fibers, and collagen fibrils from the digital images were measured using iTEM.

## Abbreviations

B: Electron-dense bands
BM: Basement membrane
BW: Bowman’s layer
EC: Elastic core
CF: Collagen fibril
CLJ: Corneolimbal junction
DM: Descemet’s membrane
DME: Descemet’s membrane endpoint
E: Elastic fiber
EL: Elastic-like fibers
EN: Endothelium
F: Fibrils
F1: Fibrils with a thickness of 16.13 ± 4.24 nm
F2: Fibrils with a thickness of 10.14 ± 2.70 nm
KR: Keratocyte
LU: Lucent space
PENL: Pre-endothelial structure (fibrous layer at the anterior side of the endothelium)
MF: Microfibrils
PDL: Pre-Descemet’s membrane layer
S: Stroma
SH: Sheath material consisting of electron-dense bands (narrow banded fiber)
TB: Trabecular meshwork
WF: Wide-spacing fibers
